# The concentration data of heavy metals in vegetables of Guilan province, Iran

**DOI:** 10.1016/j.dib.2018.10.114

**Published:** 2018-11-03

**Authors:** Dariush Naghipour, Mohsen Abbasi Chenari, Navid Taheri, Fatemeh Naghipour, Fardin Mehrabian, Mir Saeed Attarchi, Jalil Jaafari, Esmail Roubakhsh

**Affiliations:** a School of Public Health, Guilan University of Medical Sciences, Rasht, Iran; b Guilan University of Medical Sciences, Rasht, Iran; c Department of Environmental Health, School of Public Health, Tehran University of Medical Sciences, Tehran, Iran

**Keywords:** Vegetables, Heavy metals, Daily intake

## Abstract

Food safety is a major problem currently facing the world and food consumption has been identified as the major pathway for human exposure to hazardous pollutants such as heavy metals. These datasets include the concentration of heavy metals like Cd, Pb, Cu, Ba, Co and Sn in selected vegetables in Guilan province and estimate daily intake of metals. The results of this dataset showed that the average concentration of heavy metals including Cd, Pb, Cu, Ba, Co and Sn in total vegetables were 0.55, 1.098, 4.095, 5.98, 0.69, and 0.2 mg/kg, respectively. The mint showed higher levels of Pb, Cu and Co contamination compared to other vegetables. The estimated daily intakes of Cd, Pb, Cu, Ba, Co and Sn for children were 0.311, 0.622, 2.320, 3.388, 0.391, 0.119 µg/day, whereas for adults were 0.182, 0.363, 1.357, 1.98, 0.228, 0.069 mg/kg, respectively. The present data highlights that both adults and children consuming vegetables ingest significant amount of these metals.

**Specifications table**

**Table:** 

Subject area	Chemistry, Biology
More specific subject area	Vegetables monitoring and quality
Type of data	Table, image, figure
How data was acquired	ICP-OES(Instrument Model: Varian VISTA-MPX)
Data format	Raw, analyzed
Experimental factors	Measuring the concentration of heavy metals (Cd, Pb, Cu, Ba, Co and Sn) in the samples of vegetables (Dill, Radish leaves, Radish, Parsley, Spinach, Spring onion, Leek, Fenugreek, Mint and Coriander) in Rasht city.
	After determining the concentration of heavy metals estimated daily intake dose were calculated.
Experimental features	Determine the content levels of heavy metals
Data source location	Guilan province, Rasht, Iran
Data accessibility	Data are accessible with the article
Related research article	M. Arora, B. Kiran, S. Rani, A. Rani, B. Kaur, N. Mittal, Heavy metal accumulation in vegetables irrigated with water from different sources, Food chemistry, 111 [1] 811–815.

**Value of the data**

• Data can be used as a base-line data for heavy metals concentration levels in vegetables.
• There is always the possibility of contamination of vegetables with pollutants such as heavy metals and toxic substances, therefore, continuous monitoring is essential for public health workers.
• Data shown here may serve as benchmarks for other groups working in the field of food, and toxicology to compute heavy metals daily intakes by food consumption.
• The results of this dataset can be useful for Iranian Environmental Protection Agency, Ministry of Health for human health risk assessment of vegetables product.

## 1 Data

Table 1 shows descriptive statistics of selected heavy metals in the sampled vegetables, also Table 2 shows daily intake of metals for individual heavy metals in different vegetables for both adults and children.

**Table 1:** The heavy metal contents in the sampled vegetables.

Plants	Number of samples	Metals					
		Cd	Pb	Cu	Ba	Co	Sn
Dill	10	0.44 ± 0.28	0.83 ± 0.64	2.74 ± 0.083	3.33 ± 3.56	0.38 ± 0.28	0.24 ± 0.15
Radish leaves	9	0.6 ± 0.05	1.11 ± 0.045	4.41 ± 0.09	3.37 ± 2.8	0.78 ± 0.68	0.25 ± 0.11
Radish	10	2.57 ± 3.65	1.58 ± 1.41	3.1 ± 0.02	7.46 ± 13.34	0.76 ± 0.98	0.35 ± 0.26
Parsley	9	0.34 ± 0.19	0.58 ± 0.26	2.8 ± 0.79	9.27 ± 12.4	0.36 ± 0.23	0.15 ± 0.03
Spinach	11	0.23 ± 0.08	0.94 ± 0.41	3.99 ± 0.8	3.93 ± 4.67	0.83 ± 1.56	0.19 ± 0.07
Spring onion	10	0.20 ± 0.07	0.58 ± 0.22	5.44 ± 5.19	10.06 ± 15.15	0.92 ± 0.91	0.16 ± 0.05
Leek	10	0.37 ± 0.17	1.27 ± 1.06	4.22 ± 1.51	6.07 ± 6.18	0.80 ± 0.61	0.18 ± 0.13
Fenugreek	10	0.29 ± 0.03	1 ± 0.39	3.42 ± 0.97	4.15 ± 4.94	0.58 ± 0.53	0.21 ± 0.11
Mint	10	0.2 ± 0.03	1.80 ± 0.94	6.22 ± 2.385	7.28 ± 10.25	0.89 ± 0.8	0.18 ± 0.15
Coriander	11	0.26 ± 0.09	1.29 ± 0.26	4.61 ± 1.56	4.93 ± 3.48	0.63 ± 0.43	0.19 ± 0.06

**Table 2:** Daily intake of metals for individual heavy metals in different vegetables for both adults and children.

	Cd	Pb	Cu	Ba	Co	Sn
Average concentration (mg/kg)	0.55	1.098	4.095	5.98	0.69	0.21
Estimated daily intake (µg/day) for Adults	0.182	0.363	1.357	1.98	0.228	0.069
Estimated daily intake (µg/day) for Children	0.311	0.622	2.320	3.388	0.391	0.119

## 2 Experimental design, materials and methods

### 2.1 Dataset area

**Fig. 1:**
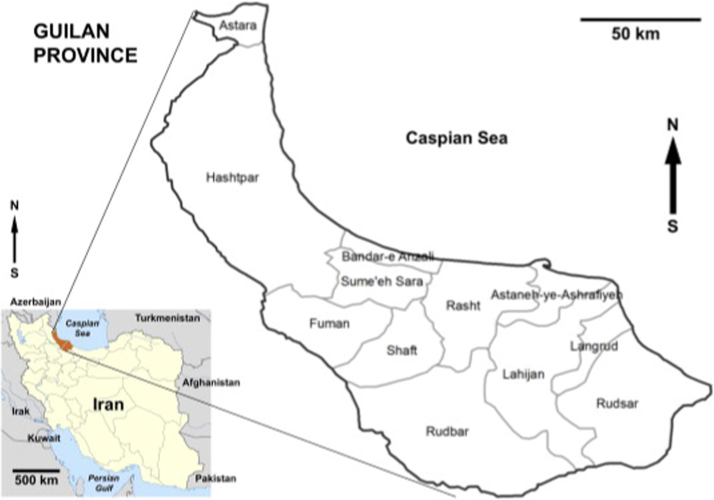
The location of the dataset area.

Guilan Province lies along the Caspian Sea, in Iran׳s Region 3, west of the province of Mazandaran, east of the province of Ardabil, and north of the provinces of Zanjan and Qazvin. It borders the Republic of Azerbaijan in the north and Russia across the Caspian Sea. The city area is about 14,042 km^2^. Based on the latest population census in Iran (2016), its population was 2,530,696 persons (Fig. 1).

### 2.2 Sample collection and analytical procedures

This data investigated the amount of heavy metal concentrations in imported vegetables to Guilan province which 100 samples were collected. One kilogram of each vegetable was bought, separately washed, air dried and hashed. One gram of each prepared samples was carefully scaled and 3 ml nitric oxide 65% was added, then was kept at room temperature during the night then warmed in 130 °C for 3 h in hot plate and after that samples were cooled. About 2.7 ml nitric oxide and 0.6 ml perchloric acid and 43 cc distilled water were added to each sample. Then, all samples were warmed in 230 °C for 3 h repeatedly and were solved in 5 ml distilled water. All the samples were measured with ICP-OES [2], [3], [4], [5]. Table 1 shows the concentration of measured heavy metals. After determining the concentration of heavy metals in vegetables, the estimated daily intake dose [6], [7], [8] was calculated according to Eq. (1).

(1) $$\mathrm{EDI}=\frac{C\times F_{\mathrm{IR}}\times k}{W_{\mathrm{AB}}}$$

Where *C* (mg kg−1) is the concentration of heavy metals in edible portions of vegetables (Cd, Pb, Cu, Ba, Co and Sn), F_IR_ (g per day) the average daily consumption of vegetables, and W_AB_ (kg) the body weight [9], [10], [11], [12].

*K*: The conversion factor used to convert fresh green vegetable weight to dry weight was 0.085, as described by Rattan et al., [13], [14]. The National Nutrition and Food Research Institute of Iran estimated the average consumption of edible vegetables is 218 g/person/day. While, the average adult and child body weights were considered to be 55.9 and 32.7 kg, respectively [15], [16]. The daily intake of metals was estimated according to the average vegetable consumption for both adults and children (Table 2).

## Figures and Tables

**Fig. S1:**
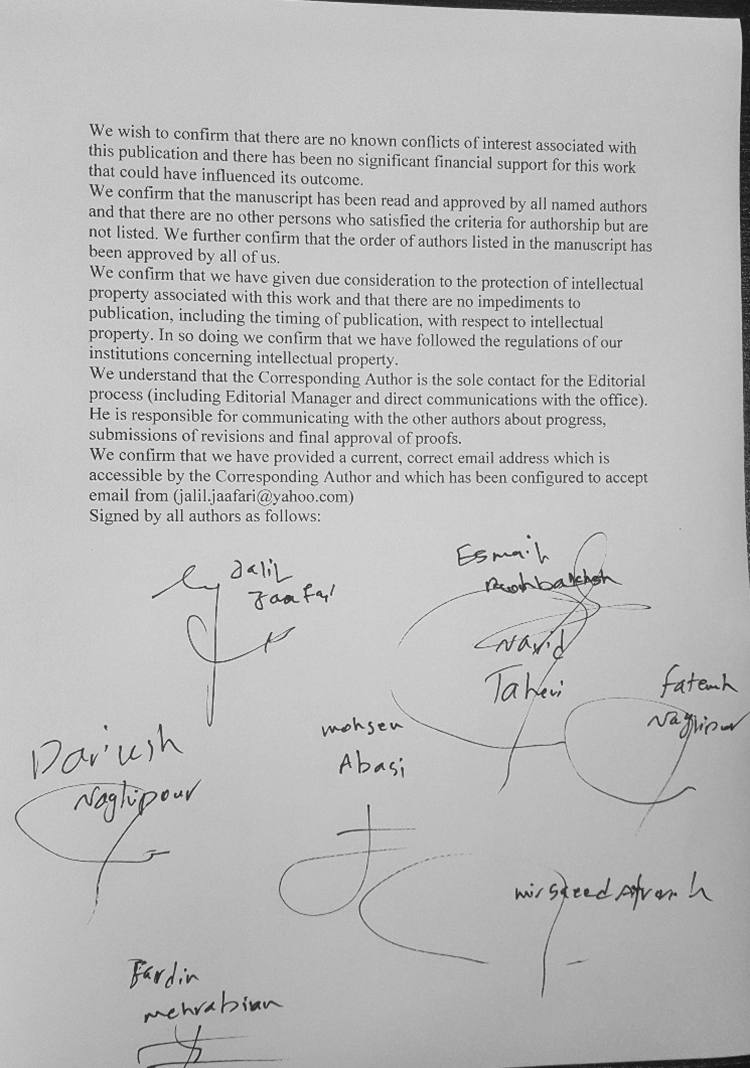


**Fig. S2:**
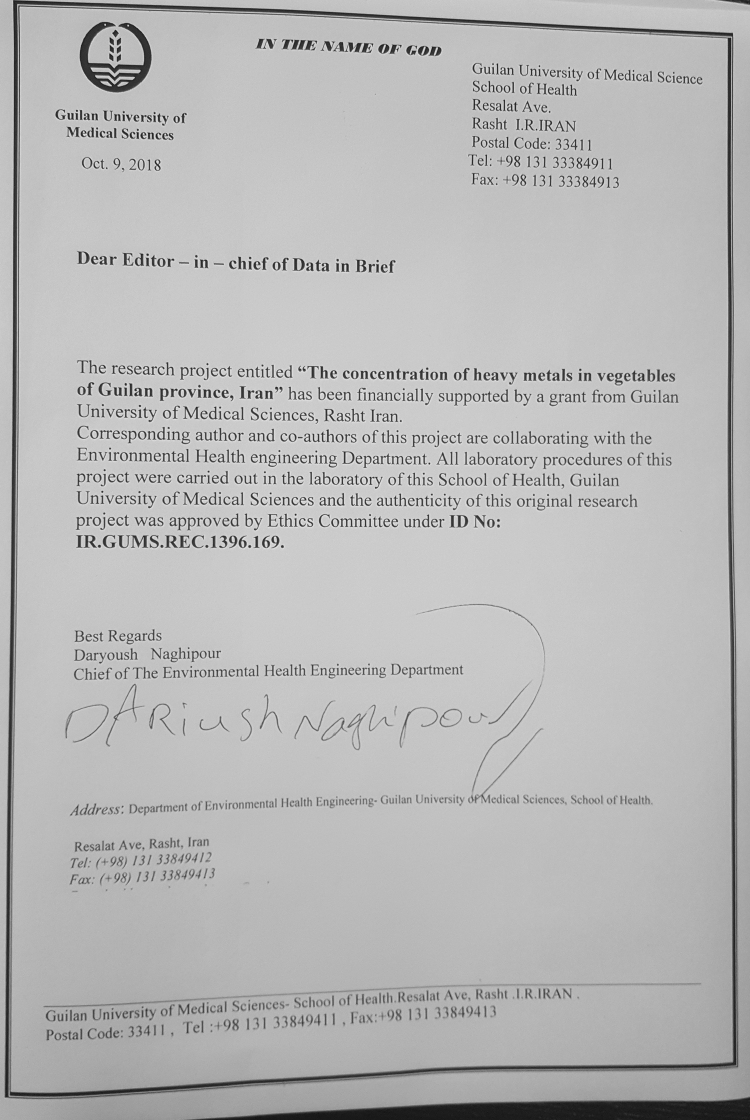


## References

[1] US Environmental Protection Agency. National Ambient Air Quality Criteria Standards. Available at: 〈http://www.epa.gov/air/criteria.html〉. Accessed September 1.

[2] Hassani S., Sepand M., Jafari A., Jaafari J., Rezaee R., Zeinali M., Tavakoli F., Razavi-Azarkhiavi K. (2015). Protective effects of curcumin and vitamin E against chlorpyrifos-induced lung oxidative damage. Human. Exp. Toxicol..

[3] Naghipour D., Gharibi H., Taghavi K., Jaafari J. (2016). Influence of EDTA and NTA on heavy metal extraction from sandy-loam contaminated soils. J. Environ. Chem. Eng..

[4] Safari G.H., Zarrabi M., Hoseini M., Kamani H., Jaafari J., Mahvi A.H. (2015). Trends of natural and acid-engineered pumice onto phosphorus ions in aquatic environment: adsorbent preparation, characterization, and kinetic and equilibrium modeling. Desalination Water Treat..

[5] Naghipour D., Taghavi K., Jaafari J., Mahdavi Y., Ghanbari Ghozikali M., Ameri R., Jamshidi A., Hossein Mahvi A. (2016). Statistical modeling and optimization of the phosphorus biosorption by modified Lemna minor from aqueous solution using response surface methodology (RSM). Desalination Water Treat..

[6] Chaturvedi R., Favas P., Pratas J., Varun M., Paul M.S. (2018). Assessment of edibility and effect of arbuscular mycorrhizal fungi on Solanum melongena L. grown under heavy metal (loid) contaminated soil. Ecotoxicol. Environ. Saf..

[7] Yousefi M., Yaseri M., Nabizadeh R., Hooshmand E., Jalilzadeh M., Mahvi A.H., Mohammadi A.A. (2018). Association of hypertension, body mass index, and waist circumference with fluoride intake; water drinking in residents of fluoride endemic areas, Iran. Biol. trace Elem. Res..

[8] Moghaddam V.K., Yousefi M., Khosravi A., Yaseri M., Mahvi A.H., Hadei M., Mohammadi A.A., Robati Z., Mokammel A. (2018). High concentration of fluoride can be increased risk of abortion. Biol. trace Elem. Res..

[9] Pirsaheb M., Fattahi N., Sharafi K., Khamotian R., Atafar Z. (2016). Essential and toxic heavy metals in cereals and agricultural products marketed in Kermanshah, Iran, and human health risk assessment. Food Addit. Contam.: Part B.

[10] Sadeghi E., Mohammadi M., Sharafi K., Bohlouli S. (2015). Determination and assessment of three heavy metal content (Cd, Pb and Zn) in Scomberomorous commerson fish caught from the Persian Gulf. Bulg. Chem. Commun..

[11] Azari A., Gharibi H., Kakavandi B., Ghanizadeh G., Javid A., Mahvi A.H., Sharafi K., Khosravia T. (2017). Magnetic adsorption separation process: an alternative method of mercury extracting from aqueous solution using modified chitosan coated Fe3O4 nanocomposites. J. Chem. Technol. Biotechnol..

[12] Dobaradaran S., Nabipour I., Saeedi R., Ostovar A., Khorsand M., Khajeahmadi N., Hayati R., Keshtkar M. (2017). Association of metals (Cd, Fe, As, Ni, Cu, Zn and Mn) with cigarette butts in northern part of the Persian Gulf. Tob. control.

[13] Rattan R., Datta S., Chhonkar P., Suribabu K., Singh A. (2005). Long-term impact of irrigation with sewage effluents on heavy metal content in soils, crops and groundwater—a case study. Agric., Ecosyst. Environ..

[14] Naghipour D., Jaafari J., Ashrafi S.D., Mahvi A.H. (2017). Remediation of heavy metals contaminated silty clay loam soil by column extraction with ethylenediaminetetraacetic acid and nitrilo triacetic acid. J. Environ. Eng..

[15] Arora M., Kiran B., Rani S., Rani A., Kaur B., Mittal N. (2008). Heavy metal accumulation in vegetables irrigated with water from different sources. Food Chem..

[16] Jaafari J., Seyedsalehi M., Safari G., Arjestan M.E., Barzanouni H., Ghadimi S., Kamani H., Haratipour P. (2017). Simultaneous biological organic matter and nutrient removal in an anaerobic/anoxic/oxic (A 2 O) moving bed biofilm reactor (MBBR) integrated system. Int. J. Environ. Sci. Technol..

